# Direct investigation of the atomic structure and decreased magnetism of antiphase boundaries in garnet

**DOI:** 10.1038/s41467-022-30992-3

**Published:** 2022-06-09

**Authors:** Kun Xu, Ting Lin, Yiheng Rao, Ziqiang Wang, Qinghui Yang, Huaiwu Zhang, Jing Zhu

**Affiliations:** 1 National Center for Electron Microscopy in Beijing, School of Materials Science and Engineering, The State Key Laboratory of New Ceramics and Fine Processing, Key Laboratory of Advanced Materials (MOE), Tsinghua University, Beijing, 100084 P.R. China; 2 Ji Hua Laboratory, Foshan, Guangdong, P.R. China; 3 Central Nano & Micro Mechanism, Beijing, Tsinghua University, Beijing, 100084 P.R. China; 4 Department of Chemical and Biological Engineering, The Hong Kong University of Science and Technology, Clear Water Bay, Hong Kong SAR, P.R. China; 5 State Key Laboratory of Electronic Thin Films and Integrated Devices, University of Electronic Science and Technology of China, Chengdu, 610054 P.R. China; 6 Hubei Yangtze Memory Laboratories, Wuhan, 430205 P.R. China

**Keywords:** Magnetic properties and materials, Surfaces, interfaces and thin films, Transmission electron microscopy

## Abstract

The ferrimagnetic insulator iron garnets, tailored artificially with specific compositions, have been widely utilized in magneto-optical (MO) devices. The adjustment on synthesis always induces structural variation, which is underestimated due to the limited knowledge of the local structures. Here, by analyzing the structure and magnetic properties, two different antiphase boundaries (APBs) with individual interfacial structure are investigated in substituted iron garnet film. We reveal that magnetic signals decrease in the regions close to APBs, which implies degraded MO performance. In particular, the segregation of oxygen deficiencies across the APBs directly leads to reduced magnetic elements, further decreases the magnetic moment of Fe and results in a higher absorption coefficient close to the APBs. Furthermore, the formation of APBs can be eliminated by optimizing the growth rate, thus contributing to the enhanced MO performance. These analyses at the atomic scale provide important guidance for optimizing MO functional materials.

## Abstract (web summary)

Iron garnets are widely used in magneto-optical devices, but knowledge of the effects of common defects on performance is limited. Here, using high-resolution microscopy and spectroscopy, the authors find that magnetism is weakened near these defects causing reduced performance, but can be avoided by tuning the growth rate.

## Introduction

Iron garnet materials have been extensively utilized in magneto-optical (MO) devices because of their high thermal strength, high Verdet constants, and low optical absorption coefficient^1,2^. In particular, yttrium iron garnet Y_3_Fe_5_O_12_ (YIG) exhibits low spin-wave damping, leading to spin-wave propagation with over centimeter distances^3–5^. Moreover, this ferrimagnetic insulator has the narrowest known line of ferromagnetic resonance (FMR), resulting in a magnon lifetime of a few nanoseconds^4^. Therefore, because of their unique linear and nonlinear spin-wave dynamics, these materials have been widely used as microwave devices such as high-*Q* microwave oscillators, filters, generators, and power limiters)^6,7^. In addition, the production of spin-wave waveguides composed of thin-film YIG having high quality makes it possible for spin waves and spin-wave dynamics to be studied. Furthermore, based on the availability of low-damping iron garnet waveguides, many spin-wave-based analogue signal processing devices have been developed. Iron garnets also attract a large amount of attention because of the high figure of merit of MO properties. Much effort has been given towards enhancing MO properties by designing specific element compositions in different cation sites for the garnet structure^8–10^. Among these specific compositions, bismuth and rare earth elements such as cerium were chosen as effective substituents to increase the Faraday rotation angle by several magnitudes compared with YIG, to attain the material suitable for integrated MO devices^9,11^. Due to the strong coupling effects among multiple order parameters, including lattice, charge, spin, and orbital, the introduced substituent in the garnet structure would change the local lattice and electronic structure, which further influences the exchange interaction between different cation sites.

To achieve the adjustable composition of garnet that can be tailored for specific applications, garnet films epitaxially grown on different substrates with high qualities have been prepared by different synthetic methods, such as liquid phase epitaxial (LPE)^11^. However, because components for preparation exhibit different chemical and physical stabilities, the adjustment of growth conditions, for example, such as temperature, growth rate, and atmosphere, can always induce some unexpected defects or impurities. These impurities, such as Bi_2_O_3_ or CeO_2_, can decrease the single crystallinity and exhibit almost no MO response. Meanwhile, impurities in large sizes can always induce cracks in the surface of films, which degrades the stability and tolerance of the incident laser, thus impairing functionalities^12,13^. For example, as candidates for MO devices with high laser-induced-damage threshold (LIDT), element substituted garnet films prepared with different synthetic methods or heat treatment exhibit significantly different properties^8,11,14^. Although experimental evidence shows that structural variation or defects induced by the fluctuation of growth conditions lead to increased or decreased performance in fabricated garnet structure^13,15,16^, investigations on the atomic structure for these defective structures, combined with the revelation of their hidden coupling effect between multiple order parameters, have been rarely discussed. Therefore, studying the defects and structural variation in the prepared garnet films should be of great significance for optimizing the MO material performance.

Antiphase boundaries (APBs) have been observed and studied in various oxide functional materials such as Fe_3_O_4_, LiFePO_4,_ and oxide heterojunctures^17–19^. However, the effects of APBs on functionality have not been revealed unless the interfacial structure was well understood^17^. It is worth noting that APBs formed in Fe_3_O_4_ can induce antiferromagnetic coupling between adjacent domains and lead to a lower spin polarization for integrated devices. Based on the knowledge of APBs, some strategies are proposed and applied to design APBs artificially in magnetic oxide materials, where these APBs have an intriguing antipolar structure phase^19^. Many studies have focused on and designed APB-related chemical and physical properties^20,21^, but the effect of APBs is still unclear due to the limited knowledge of their related atomic structures in various oxide materials. Thanks to the advancement of electron microscopy with aberration correction capability^22^, the measurement at high spatial resolution enables the atomic structure variation, element distribution, and site-specific orbital and magnetic information to be unveiled^23^.

In this study, by using atomic resolution transmission microscopy combined with energy-dispersive X-ray spectroscopy (EDS) and electron energy loss spectroscopy (EELS), two individual types of APBs have been observed in Tm_2.28_Bi_0.72_Fe_4.3_Ga_0.7_O_12_ (TBIG) (where Tm is Thulium) prepared with a growth rate of 1.00 μm/min. In APB-I, two adjacent regions shift against each other by 1/4[010] vector, but the interfacial structure reconstructs through a zigzag arrangement, which maintains the sequential arrangement of cation sites. Unlike APB-I, in APB-II, due to the extension of dislocations, two adjacent regions tend to shift against each other by 1/8[120] vector. This additional shift along the [100] direction leads to a complex interfacial structure distinctly different from that of APB-I. Moreover, element-specific magnetic information around local APB regions has been rarely investigated due to the limited resolution. By using the latest developed electron magnetic circular dichroism (EMCD) technique with high spatial resolution and element specificity, it is revealed that the magnetic signals detected when approaching the APBs decrease significantly, indicating the weakened magnetism near the APB interface compared with the internal region. Furthermore, the EELS in atom resolution confirms the segregation of oxygen deficiencies across the APBs, which can account for the decreased magnetism as well as for the high absorption coefficient of incident light. Furthermore, after the radiation of the argon (Ar^+^) ion beam, these APBs tend to become amorphous, thus implying that APBs in iron garnets could be less stable when they suffer from radiation as MO devices. Thus, to enhance the MO performance of films, the growth rate has been optimized to decrease to around 0.84 µm/min. As a result, the APBs were rarely observed in the epitaxial films. The ability to gain insight into the atomic structure can provide useful guidance to understand the relationship between the properties and structure of MO materials and devices. Therefore, it can pave the way to optimize the growth condition for MO functional materials.

## Results

### The atomic structure analysis of the APBs

**Fig. 1:**
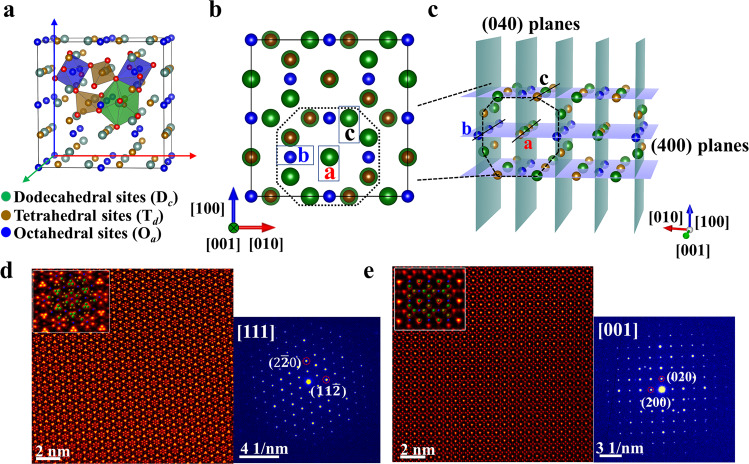
Atomic structure of unit cells in iron garnet films. **a** The atomic model of garnet in the unit cell. **b** The projected atomic arrangement along the [001] zone axis. **c** In-depth view of the unit cell along the [001] axis. Atomic columns labeled as a and c are occupied by dodecahedral and tetrahedral sites, while the one labeled as b is occupied only by octahedral sites. **d**, **e** The STEM-HAADF images of films in defect-free region are obtained along the [111] (**d**) and [001] (**e**) zone axes, where insets in an enlarged view extracted from HAADF images are consistently overlapped with the atomic model. On the right sides, the selected area of the electron diffraction patterns in the prepared film is acquired along corresponding zone axes.

The iron garnet structure has a complex cubic structure (space group as ${{{{\rm{O}}}}}_{h}^{10}-{Ia}\bar{3}d$), with a composition, expressed as {R_3_}_c_[Fe_2_]_a_[Fe_3_]_d_O_12_ (R is referred to as cation with a large radius). As shown in Fig. 1a, the cations with a large radius will be situated in dodecahedral (*c*) sites, while the Fe^3+^ and other cations with a smaller radius occupy either tetrahedral (*d*) or octahedral (*a*) sites in the oxygen polyhedron structure. These crystallographic sites are bridged between each other via oxygen atoms, for which the magnetic ions in tetrahedral and octahedral sites are antiferromagnetically coupled by the super-exchange interaction^24^. Figure 1b depicts the projected atomic arrangements viewed from the [001] zone axis. Dodecahedral and octahedral sites are overlapped in positions a and c (Fig. 1c), while position b is only occupied by octahedral sites. Here, the TBIG prepared by the LPE method exhibits a perfect garnet structure as shown in the high angle annular dark field (HAADF) images (Fig. 1d, e). When viewed from the [111] zone axis, the atomic columns in the dodecahedral sites have brighter contrast compared to those in the octahedral and tetrahedral sites. The observed atomic arrangements viewed from the [001] zone axis present the periodical lattice structure consistent with standard garnet. It is well established that the intensity of the scanning transmission electron microscopy (STEM) HAADF image (known as a *Z*-contrast image) is approximately proportional to the square of atomic numbers^25^. The HAADF images show that the Tm and Bi elements with a larger radius mainly occupy dodecahedral sites with higher contrast, while lighter elements like Fe and Ga mainly occupy octahedral and tetrahedral sites with lower contrast. Moreover, X-ray diffraction (XRD) (see Supplementary Fig. 1) and selected area diffraction patterns along the [111] and [001] zone axes in the prepared film show obvious garnet structure without any additional superlattice spots, indicating that there is no ordered distribution of substituted elements in the three different sites. Therefore, it can be concluded that the substituted elements of Bi and Ga with minor concentrations are randomly distributed in these sites.

**Fig. 2:**
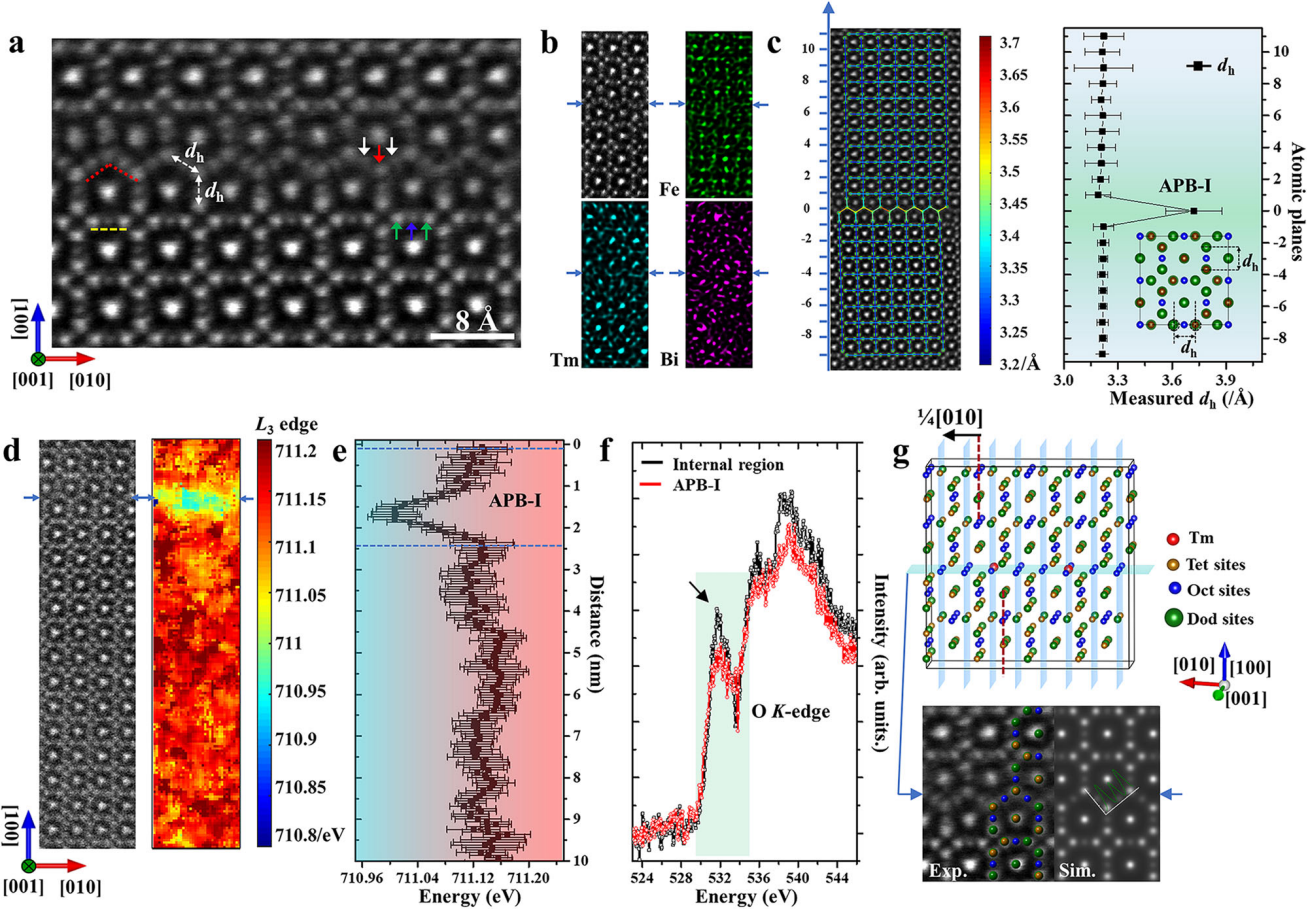
The interfacial structure and composition analysis of APB-I. **a** STEM-HAADF images of APB-I along the [001] zone axis. Green arrows represent atomic columns where dodecahedral sites and tetrahedral sites overlap each other (while positions indicated by blue arrows are occupied by octahedral sites). Red lines highlight the interfacial atomic structure like a zigzag pattern, where atoms marked by red and white arrows are distinguished with different contrast. **b** The corresponding STEM-HAADF image and EDS colored maps of Tm, Bi, and Fe elements. The intensity of color represents the concentration of elements. The interfacial region is indicated by blue arrows, where signals of the Tm element are found along a zigzag line structure. **c** The spacing *d*_h_ defined as shown in (**a**) is measured and compared across the APBs. The error bar is defined by the standard deviation of measured spacing *d*_h_. **d** The EEL spectra at the atomic scale are acquired across the interfacial region. Fe *L*_3_ edges energy positions are extracted from the EELS maps, as shown in the color map. **e** The quantitative profile of the Fe *L*_3_ edge positions is compared across the APBs. The error bar is defined by the standard deviation of measured Fe *L*_3_ edge positions. **f** EEL spectra of the O-*K* edges are acquired in different regions. **g** Based on the previous analysis, the proposed atomic model of APB-I is presented. The simulated HAADF images based on the proposed model are overlapped in the STEM-HAADF image. The line profile intensity (inset) extracted from interfacial atom columns is analogous to the results of the experimental image.

As it has been recognized that the prepared films with the composition of TBIG belong to the phase with garnet structure, two typical kinds of APBs (APB-I and APB-II) were first discovered by the authors of the present study in this complex garnet structure when closely examining the areas with boundaries. For APB-I, two adjacent regions shift against each other by 1/4[010] vector (Fig. 2a). This unexpected variation breaks the local symmetry and can lead to the reconstructed interface as a transitional area between the adjacent regions^26,27^. The STEM-HAADF image in Fig. 2a shows that the two adjacent regions are connected with a sharp interface, but the interfacial structure reconstructs through a zigzag atomic arrangement. Atomic columns marked by red and white arrows are alternately well accommodated with the upper or lower atomic columns to form a periodical zigzag structure highlighted by red polygonal lines. Away from the interface, atomic columns marked by green arrows are occupied by dodecahedral and tetrahedral sites, and those marked by blue arrows are occupied by octahedral sites. To maintain the structural continuity across the APBs, it can be inferred that atomic columns marked by red arrows are occupied by mixed sites of dodecahedron and tetrahedron and the ones that are marked by white arrows are occupied by octahedral sites (see Supplementary Fig. 2c). Therefore, these atomic arrangements along the interface can maintain the connection of the interfacial structure for the diverse polyhedral network. Besides, the intensity profile of atomic columns corresponding to the internal area and the near-interface area in HAADF images are plotted (Supplementary Fig. 3), showing that the atomic contrast in the zigzag structure marked by red lines along the interface is inverse, which is compared with that marked by yellow lines in the region away from the interface.

Direct EDS maps at the atomic scale (Fig. 2b) show that the Fe element is randomly distributed across the APBs and the Tm element is mainly occupied in dodecahedral sites, though tetrahedral sites are overlapped with dodecahedral sites along the [001] zone axis. To check the variation of elements across the interface, the integrated EDS line profile intensities for different elements (Supplementary Fig. 3d) were plotted and it was found that Bi and Ga concentration is limited as a minor substituent. No Fe and Ga segregation or loss exists in the region near the interface. However, along the interface, the EDS signal of the Tm and Fe elements is prominently observed, indicating that partial octahedral sites marked by white circles (Fig. 2a) are occupied by Tm^3+^ cations. Similar unexpected site exchanges in garnet have also been reported in previous related works^28,29^, where cations with a larger radius like Y^3+^, which are supposed to be located in dodecahedral sites, were revealed to occupy octahedral sites, known as the antisite defect^30^. Due to the smaller effective cation radius (0.994 Å) of Tm^3+^ compared with Y^3+^ (1.019 Å)^31^, Tm^3+^ possibly occupies octahedral sites if the synthetic parameters for film preparations are varied. In addition, the defined spacing as *d*_h_ of adjacent atoms (shown in Fig. 2c) in the zigzag interfacial structure is measured along the APB-I for comparison. The space between the adjacent atoms in the interface of the zigzag structure increases, which is larger than that of the normal region away from the interface. Therefore, the enlarged space of the zigzag structure in the interface can accommodate trivalent cations with a larger ionic radius. Furthermore, this antisite behavior is confirmed through direct element distribution maps by EDS (Supplementary Fig. 4) in the internal region close to the APB-I. Obvious EDS signals of Tm are observed in these octahedral and tetrahedral sites. This antisite behavior is often observed in the sites for cations with a similar radius^28,29^. However, it is not observed for Bi elements, which is due to the larger radius of Bi^3+^ (Bi^3+^: 1.17 Å)^31^ compared with that of Y^3+^.

Furthermore, atomic-column resolved EELS is performed to determine the element distribution and oxide state of cations in different sites^23,32,33^. Energy positions of the Fe *L*_3_ edges are mapped across the APBs, as shown in Fig. 2d. The lower energy positions of the Fe *L*_3_ edges close to the APBs indicate the lower valence state of the Fe ions (Fig. 2e), which can be attributed to the oxygen deficiencies^34,35^. It can be reasonably assumed that the enlarged space across the APB-I weakens the bonding strength of the Fe-O polyhedral sites, leading to the extra oxygen vacancies^36–39^. The EELS map in an enlarged view of APB-I (Supplementary Fig. 5a) further reveals that the oxygen deficiencies tend to segregate along the APBs. In addition, a few spectra of Fe, O, and Tm extracted from the APB interface and the region away from APB are compared. The O-*K* edge is strongly related to the O 2*p* orbital hybridized with other elements (Fig. 2f)^22^. The decreased strength of the pre-peaks marked by the black arrow indicates an increase in oxygen vacancies. It can be found that the strength of the pre-peak in APBs decreases compared with that in the internal region, thus confirming that the reduced valence state of Fe across the APBs is induced by oxygen vacancies. The weak signal of Tm *M*_5_ edge can be resolved in the position marked by the red line (Supplementary Figs. 5b), which further verifies the antisite behaviors between the octahedral and dodecahedral sites.

Based upon the structural, compositional, and valence analyses, the interfacial structure is proposed as depicted in Fig. 2g, where octahedral sites in the interfacial plane have been randomly substituted by a minor amount of the Tm element marked by red atoms. The simulated STEM-HAADF image based on the proposed interfacial structure model is consistent with the experimental result. Meanwhile, the STEM-HAADF image of the interfacial structure (Supplementary Fig. 3e) along the [111] zone axis was also acquired, which is consistent with the projected atomic structure by use of the proposed model along the [111] zone axis. This evidence demonstrates the accuracy of the proposed interfacial structure and further indicates that the sites of different cations reconstruct in a zigzag line to maintain the lattice continuity along the interface of APB-I.

**Fig. 3:**
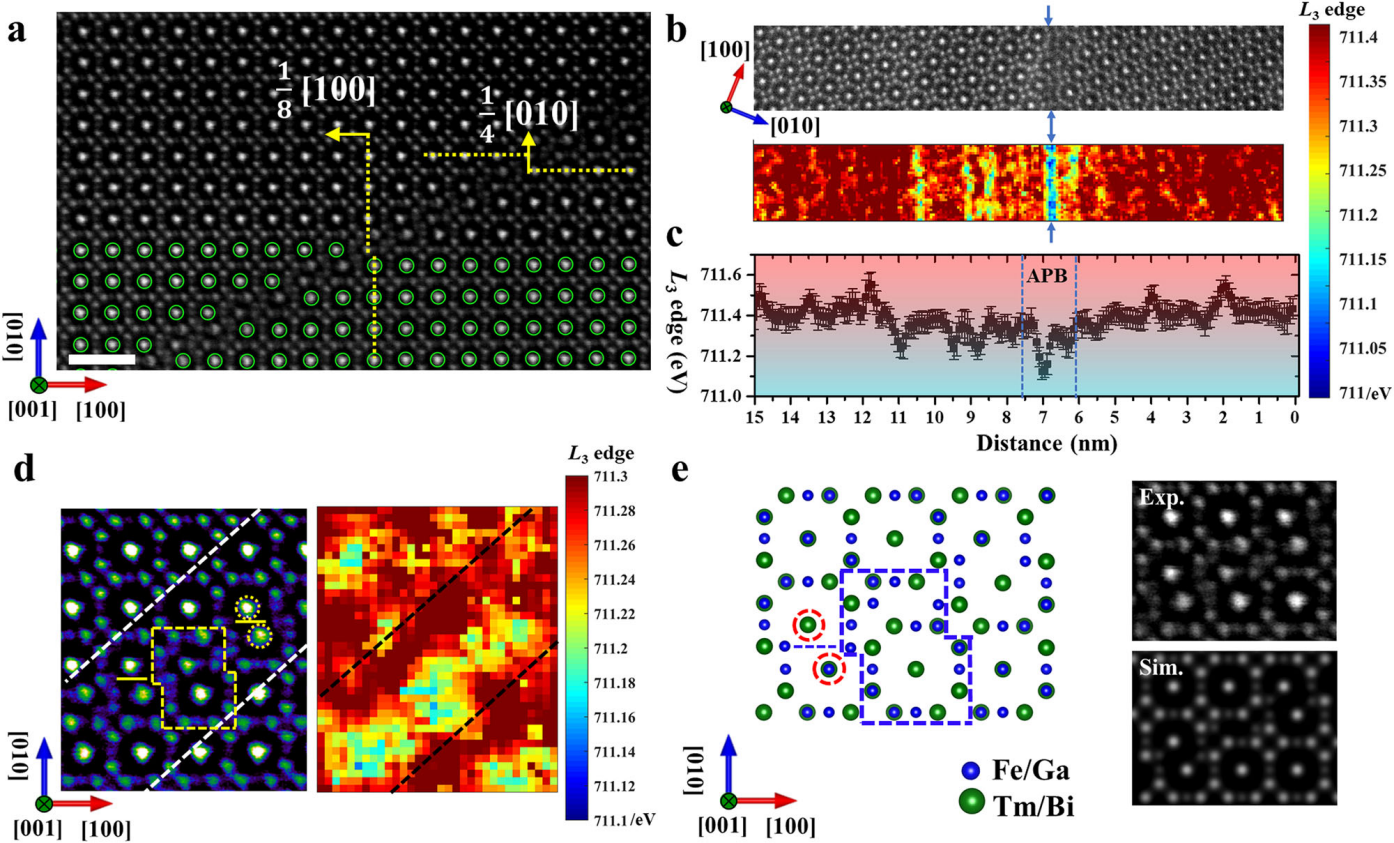
The interfacial structure and composition analysis of APB-II. **a** STEM-HAADF image of APB-II along the [001] zone axis, where the scale bar is 1 nm. **b** EELS map for Fe is acquired across the interface. Fe *L*_3_ edges energy positions are extracted from the EELS maps, as shown in the color map. **c** The quantitative profile of the Fe *L*_3_ edge positions is compared across the APBs. The error bar is defined by the standard deviation of measured Fe *L*_3_ edge positions. **d** The magnified STEM-HAADF image from (**a**). On the right side, the corresponding map for Fe *L*_3_ edge positions is mapped in the interface of the APB-II. **e** The proposed interfacial structure model is shown on the left side. The enlarged view of the interfacial structure and corresponding simulated STEM-HAADF image based on the proposed model are respectively shown on the upper and lower right sides.

Figure 3a shows another type of individual interfacial structure of APB-II, which is distinctly different from APB-I. Two adjacent regions tend to shift against each other along the [120] direction by 1/8[120] vector. The additional shift along the [100] direction by 1/8 [100] vector compared with APB-I leads to the step-like periodical structure. it can be found that the adjacent domains have different shifting distances along these two directions, thus this complex sloped shifting can be decomposed into two components (seen in Supplementary Fig. 6). The adjacent domains shift against each other by 1/4[010] unit cell along the [010] direction, while the adjacent domains shift against each other by 1/8[100] unit cell along the [100] direction. When two adjacent domains shift against each other along 1/4[010] together with 1/8[100], it can lead to the additive shifting by 1/8[120], which is equal to 1/4[010] plus 1/8[100]. Moreover, analogous to APB-I, two adjacent regions shift against each other by 1/4[010] vector, which is indicated by atoms marked with green circles. However, the interfacial structure is more complicated in APB-II and different from that in APB-I. Energy positions of the Fe *L*_3_ edge are mapped across the APB-II, where the lower valence state of Fe is also observed close to the APBs. Figure 3b, c also reveals that oxygen deficiencies tend to segregate in the APBs. Compared with the result in APB-I, the distribution of oxygen deficiencies tends to extend a few nanometers beyond the boundaries of APB-II, which can be attributed to the larger strain induced by the formation of APB-II when compared with that of APB-І (the detailed strain comparison for the two APBs can be seen in Supplementary Fig. 6). Therefore, due to the larger strain state, the distribution of oxygen deficiencies can be extended over a few nanometers across the distorted regions in APB-II^40,41^. The sloped interface is constructed with a periodical structure marked by the orange polygon and merged sublattice marked by orange circles along the [120] direction (see the left side of Fig. 3d). As shown in the orange polygon, the planes <400> of two adjacent regions stack closely, while several atomic chains (indicated by orange horizontal lines) between the two brightest atoms (marked by orange circles) are removed, leading to the merging of the two sublattices. As for the merging of the adjacent sublattice discussed above, the EELS maps of Fe and O show the decreased intensity of the EELS signal in the interface (Supplementary Fig. 8). Moreover, the lower valence state of Fe can be observed in these merging unit cells, indicating that oxygen deficiencies are clustered in these modified interfacial structures (see the right side of Fig. 3d).

To directly illustrate the interfacial structure of APB-II, an atomic structure model based on the above structure and composition analysis is proposed in Fig. 3e, where adjacent regions shift against each other, leading to the merging of two sublattices and a stacked interfacial structure marked by red circles and blue polygonal lines. Based on the proposed atomic model, the simulated STEM-HAADF image of the interfacial structure shows a close match with the experimental image, confirming the proposed interfacial model. Furthermore, the STEM-HAADF image along the [011] zone axis shows the atomic arrangement consistent with the proposed interfacial model, which demonstrates the accuracy of the proposed model (Supplementary Fig. 8). Combined with the geometric phase analysis (GPA), the strain investigation on the local defects indicates that the formation of APBs is strongly coupled with local defects to accommodate the strain filed relaxation (Supplementary Fig. 7). In particular, the segregation of oxygen vacancies across the APBs can diffuse or accumulate when the strain state varies in the boundaries^40–42^.

### The magnetic analysis of the regions close to the APBs

**Fig. 4:**
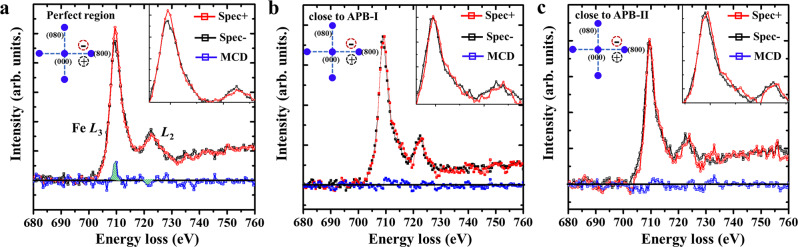
EMCD measurement in different regions. **a**–**c** The EMCD spectra (blue lines) are acquired at the perfect region, and positions corresponding to a ~2–3 nm distance from the interfaces of APB-I and APB-II, respectively. The EEL spectra recorded from the conjugated positions labeled as positive red circles and negative black circles are normalized. Then, the EMCD spectra (blue lines) can be obtained through subtraction between the positive and negative spectra (red and black lines).

**Fig. 5:**
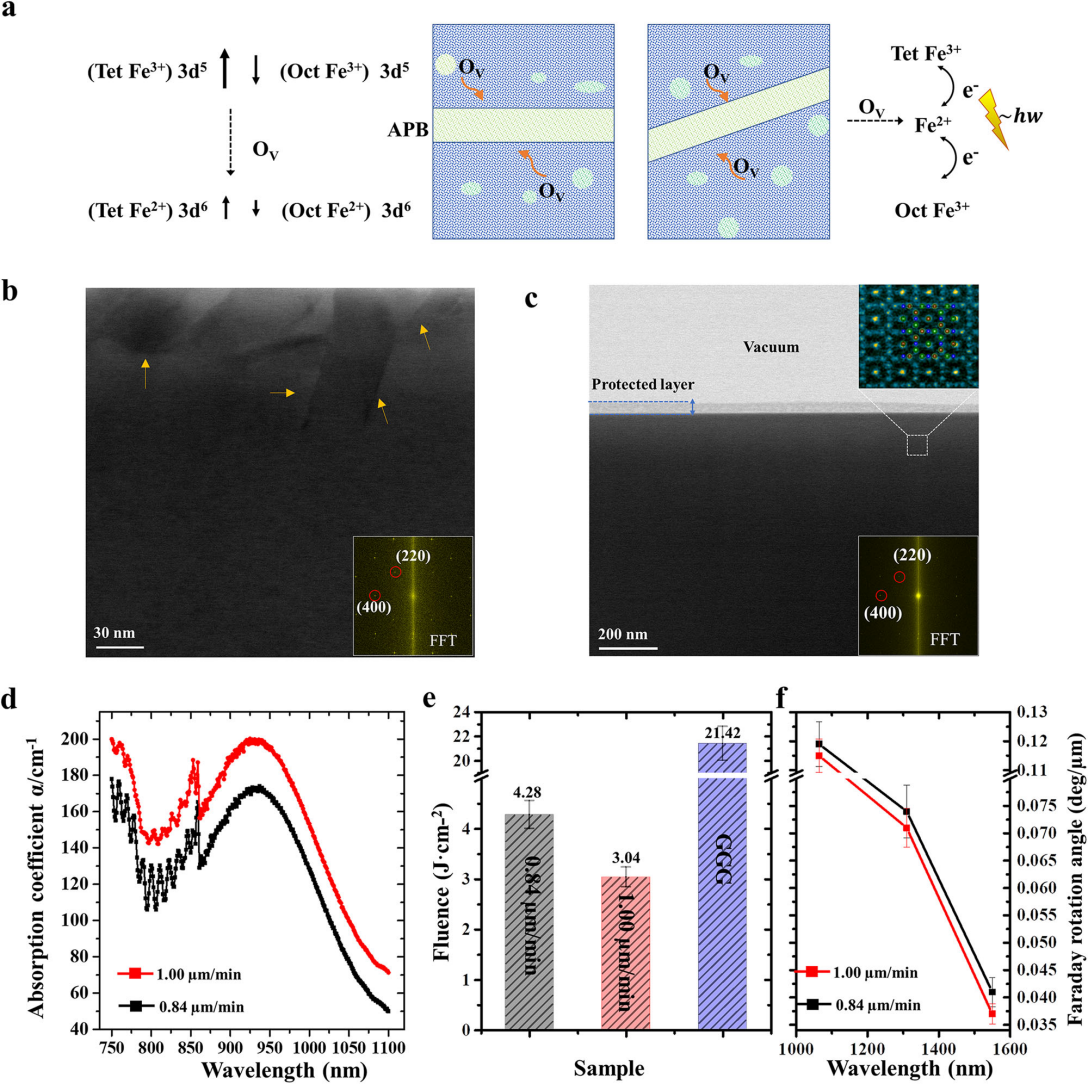
Effects of magnetic elements with a lower valence state on MO properties. **a** The schematic diagram shows that regions with APBs exhibit a decreased net magnetism and enhanced light absorption, this is because the Fe^3+^ in different sites can be reduced to Fe^2+^ due to the oxygen deficiencies. O_V_ is referred to the oxygen vacancies, and green dots represent the oxygen vacancies. Green rectangle and parallelogram indicate the APBs. **b** STEM-bright field (BF) image of the film with a larger growth rate ~1.00 µm/min, yellow arrows mark the APBs. **c**. STEM-BF image of the film in low magnification with a growth rate of 0.84 µm/min, while films prepared with a growth rate of 0.84 µm/min show perfect crystal structure without any obvious boundaries. An enlarged view extracted from the region marked by cubic lines in (b) shows perfect atomic arrangement as an atomic model along the [001] zone axis. Insets in (**b**) and (**c**) are Fast Fourier transform (FFT) images, where diffraction planes are highlighted by red circles. **d** Plots of the absorption coefficient as the function of wavelength are compared between samples prepared with different growth rates. **e** The histogram shows the comparison of the LIDT between the sample prepared with different growth rates and GGG substrates. **f** Plots of Faraday rotation angles as the function of wavelength are compared between samples prepared with different growth rates. The error bar is defined by the standard deviation of measured Faraday rotation angles.

It has been well established that MO properties are strongly related to the magnetic properties of MO materials, where they are almost linearly proportional to the Verdet constant^43,44^. Therefore, to give insight into the local magnetic behaviors around these APBs, EMCD experiments with high spatial resolution and element specificity were performed in the present study. Analogous to the well-established X-ray magnetic circular dichroism (XMCD), two conjugated positions, where collection apertures (indicated by red and black circles as shown in Fig. 4a–c) are placed, where they are chosen to acquire energy loss spectra that are respectively labeled as positive and negative spectra. Then, the EMCD signal marked by blue lines can be obtained through the difference between the positive and negative spectra. When the electron beams were located in the internal regions and positions of ~2–3 nm away from the interfaces of APBs respectively, a series of EELS can be recorded in pairs (the details of the EMCD experiment can be seen in Supplementary Figs. 9 and 10). The EMCD spectra acquired in the internal defect-free region (Fig. 4a) show an apparent positive signal in the Fe *L*_3_ edge and negative signal in *L*_2_, while the EMCD spectra in Fig. 4b, c acquired in the regions close to APBs show an almost disappeared signal in the Fe *L*_2,3_ edge. To confirm this confidence level of EMCD signals in different regions, the Gaussian fitting method is applied to fit the signal and noise value of different EMCD spectra^45^. According to Rose criterion^46^, the signal to noise ratio (SNR) for Fig. 4a exhibits an apparent EMCD signal, while Fig. 4b, c exhibit apparently decreased signals in the EMCD spectra (details can be seen in Supplementary Note 1). The intensity of the EMCD signals varies corresponding to the change in magnetic parameters for the investigated regions^47,48^. Together with macroscopic magnetization measurement (seen in Supplementary Fig. 11), this comparison indicates that the regions close to APBs show obvious decreased magnetic moments of Fe compared to the internal region. As for the ferrimagnetic magnetic structure of the iron garnet, magnetic moments of tetrahedral sites are antiferromagnetically coupled with that of octahedral sites and dodecahedral sites, which can be described as [↑Tet↓Oct↓Dod] in the unit cell for the garnet structure. However, the MO performance can be simply seen as proportional to the net magnetic moments in iron garnets^43,44^. Thus, the observed suppressed magnetic signal indicates that the formation of APBs will decrease the MO performance of iron garnet films. The decreased magnetic signal in the regions close to the APBs is strongly related to the local reduced valence state of magnetic elements^49–51^, which are caused by oxygen deficiencies, as discussed above. The oxygen element is critical for the bridge between Fe elements and plays an important role in the super-exchange interaction to maintain magnetic ordering^52–54^. As shown in Fig. 5a, the reduced Fe^2+^ can decrease the magnetic moments, since the spin moment of 3*d*^6^ (Fe^2+^) is smaller than that of 3*d*^5^ (Fe^3+^)^55–57^. Moreover, together with the observed antisite behaviors between Fe sites and dodecahedral sites, these can also weaken the Fe_oct_-O-Fe_tet_ exchange interaction^54^, as well as simultaneously decrease the Fe magnetic moments. The density function theory (DFT) calculation demonstrates that the calculated magnetic moments, bond lengths, and angles in these Fe sites with specific oxygen vacancies (6.25%) show larger bonding lengths and decreased angles than that of a perfect structure without oxygen vacancies. Compared with the perfect iron garnet structure without oxygen vacancies, the magnetic moments noticeably reduce when oxygen vacancies are introduced (details can be seen in Supplementary Table 1). Moreover, when the incident light passes through the sample, the reduced Fe^2+^ caused by the oxygen deficiencies can lead to the extra electronic transitions between Fe^2+^ and Fe^3+^ complex (as shown in Fig. 5a), thus resulting in enhanced light absorption^56,58,59^. As shown in Fig. 5d, the prepared films with APBs exhibit a larger absorption coefficient than those without obvious APBs. To some extent, compared with normal regions, these regions including APBs are favorable to the start damages because the enhanced absorption of incident light with higher energy density could significantly lead to the disruption of local ionic bonds and increase local temperature. In addition, after being irradiated by the argon (Ar^+^) ion beam, the APBs were easily transformed to be amorphous, indicating less stability compared with the bulk region (see Supplementary Fig. 12)^60,61^.

### The magneto-optical analysis of the prepared iron garnet films

Furthermore, the LPE growth condition has been optimized to enhance the quality of the film, where the control of the growth rate is discussed in a previous study^13^. Figure 5b, c shows the STEM-bright field (STEM-BF) image of the film with either a larger or an appropriate growth rate, respectively. The prepared film with a growth rate of 0.84 µm/min exhibits an obvious garnet structure (indicated by the FFT pattern in inset) without any visible APBs or any other defects, and the STEM-HAADF image in the inset shows an obvious perfect garnet structure. While there are large amounts of APBs (indicated by yellow arrows) in the prepared film with a larger growth rate (Fig. 5b). The difference in growth rate can lead to a discrepancy in the nucleating rate and ionic migration during the crystallization, so defects or ordered phase boundaries would be induced when thermodynamic conditions have been disturbed^62,63^. Figure 5e, f shows that films prepared at an optimized growth rate of ~0.84 µm/min exhibits higher laser-induced damage compared with films prepared at a higher growth rate, and the plots of the Faraday rotation angle at different wavelengths are also investigated to manifest a consistent result. These investigations indicate that an optimized growth rate can contribute to the enhanced MO performance. By optimizing the growth rate of the LPE method, the formation of APBs can be suppressed, providing the possibility to optimize the functionality of MO devices. Although restricted by the limited capability to model these complex interfacial structures in garnets by the DFT, quantitative comparisons of local electronic structures need to be explored further in future studies via advanced calculation analysis.

## Discussion

The local atomic structure and magnetic properties of two types of APBs observed in iron garnet grown by LPE on a gadolinium gallium garnet (GGG) substrate have been investigated by high-resolution analytical transmission electron microscopy. Largely different from reported APBs in other ferrite materials, the segregation of oxygen vacancies occurs across the APBs. Combined with the GPA, the different strain states can be compared between these regions close to APBs, where a larger strain can lead to the extended distribution of oxygen vacancy segregation. The EMCD techniques with high spatial resolution reveal the decreased magnetic signals in the regions close to APBs, indicating degraded MO performance when applied in the MO devices. Moreover, the reduced Fe^2+^ caused by oxygen deficiencies can result in higher light absorption due to extra transitions between Fe^2+^ and Fe^3+^, implying that the structure and properties of APBs are less stable when subjected to intense irradiation in the application of MO devices. In sum, the present study discovers the correlated structures and properties in iron garnet by direct observation at the atomic scale, which was hardly possible for previous works to achieve via other techniques. Furthermore, by optimizing the growth rate, the iron garnet film on the GGG substrate could be prepared free of APBs through the modulation of thermodynamic conditions. Inspired by the understanding of the atomic structure and the stability of defects in these complex functional materials, real-space analysis with high resolution can serve as a basis for the fundamental understanding of the MO properties, as well as to further improve the performance of the MO devices.

## Methods

### Preparation of iron garnet films

The films of TBIG were grown on GGG substrates (*a* = 12.383 Å). The TBIG films were prepared by the liquid phase epitaxy (LPE) method with a thickness 5–6  μm. Firstly, high purity ingredients (99.999%) were weighed at molar ratio and melted in the crucible at 1050 °C for 12 h, and then stirred for 12 h. The homogenized solution at high temperatures was cooled to be used as an oversaturated solution. The surface-cleaned substrate GGG would be fixed by a platinum wire, keeping a small angle (3–15°) with the melting surface. Then, the substrates were lowered to be immersed in the melting solution, allowing the film growth to begin. Simultaneously, the substrate rotated, and the rotation direction changed once every 3 min. Finally, the film was cleaned in hot nitric acid to remove the residual flux agent. The film’s thickness and growth rate were controlled by the growth time and temperature. The crystalline quality was characterized by high-resolution X-ray diffraction (XRD) using a Rigaku Smartlab X-ray diffractometer. The Laser-induced-damage threshold (LIDT) of the prepared iron garnet films was tested by using a Q-switched Nd: YAG single-mode laser with 11 ns pulse width and 1064 nm wavelength. The laser was focused to have a far field circular Gaussian beam (the diameter of the beam was 0.58 mm with maximum intensity). By monitoring the testing process by use of the He-Ne laser, the onsets of induced damages can be detected through microscopy with a charge-coupled device camera with the control of a computer. LIDT is specific terminology that is used to test the stability of MO films under working conditions with an incident laser. This value can be calculated with the minimum laser energy density when the sample is damaged (End_min_) and the maximum laser energy density (End_max_) before the sample is damaged. Therefore, LIDT can be expressed as (End_min_ + End_max_) / 2. The magnetic properties of films were measured using a vibrating sample magnetometer (BHC525, IWATSH, Japan). The magnetization of films was acquired by subtracting the diamagnetic signals from the GGG substrates.

### TEM sample preparation and TEM characterization

The STEM specimen was a lamella extracted from the sample by focused-ion-beam (FIB) milling in a Zeiss Auriga FIB using 5 kV 20 mA ion beam to reduce the thickness of the surface amorphization layer in the final step. The atomic structure and element mapping characterization were carried out by scanning transmission electron microscopy (STEM) in a FEI Titan Cubed Themis G2 300, which was operated at 300 kV and equipped with a high-brightness Schottky field emission gun and monochromator. A probe aberration corrector was applied to provide a spatial resolution better than 0.6 Å in the STEM mode. To identify the interfacial chemistry, element compositions were obtained by using Energy Dispersive X-ray Spectroscopy (EDS) and Gatan Imaging Filter Quantum Energy Filters for EELS analysis. The *Z* contrast imaging was conducted in high-angle annular dark field (HAADF) with a probe convergence angle of 25 mrad and an inner collection angle of ~80 mrad and an outer collection angle of ~240 mrad. The HAADF imaging was combined with the electron dispersive spectrum to analyze different element mapping, and ADF imaging combined with EELS was used to achieve atomic-scale EELS by spectrum imaging (SI). The EMCD experiments were performed under nanobeam-STEM mode using FEI Titan Cubed Themis G2 300. The size of the chosen aperture in GIF is 2.5 mm. To locate the APBs using the atomic scale and nanobeam mode, a featured region with Au with a specific shape as the protected layer was selected to locate the positions of APBs (details can be seen in Supplementary Fig. 9). In the EMCD measurements, all STEM experimental conditions and the simulation of dynamical diffraction conditions under the [001] zone axis, were optimized. In summary, the calculations consisted of two parts. Firstly, the Bloch coefficients and momentum transfer based on the specific experimental diffraction conditions were calculated using a Bloch wave software package^64^. Then, a Matlab code was implemented to calculate the dynamical coefficients corresponding to nonmagnetic and magnetic components^65^.

### TEM image analysis

The quantitative analysis of (S)TEM images was performed using the embedded programs in MacTempas. The atomic positions of the (S)TEM images were accurately determined by fitting the images’ intensity maxima with the two-dimensional Gaussian peaks fitting method. The simulation of STEM images was realized by using the multislice method embedded in the MacTEMPAS software. The Geometric phase analysis (GPA) was performed using a free Strain++ software^66^.

### Density-functional theory calculation (DFT)

The Density-functional calculations (DFT) were conducted via the use of the Vienna ab initio simulation package (VASP) to investigate rare-earth iron garnets^67^. The generalized gradient approximation (GGA)^68^ was used together with the Perdew-Burke-Ernzerhof (PBE) exchange-correlation functional for solids^69^. The projected augmented wave (PAW) method was used to mimic electron-ion interactions^70^. The localized Fe 3*d* electrons were treated with an effective Hubbard *U* = 4 eV. The magnetic configuration adopted for the calculations was that the magnetization of the rare-earth ions was chosen to be antiparallel to that of the tetrahedral Fe ions while being parallel to that of the octahedral Fe ions, which is consistent with a common belief for rare-earth iron garnets. Moreover, the energy cutoff was selected to be 500 eV, and the Monkhorst-Pack *k*-point mesh was chosen to be 3 × 3 × 3 for the 80-atom primitive unit cell.

## Supplementary information

Supplementary Information

## Data Availability

All data supporting the findings are available in the article.

## Code availability

All codes supporting the findings are available in the article.

## References

[1] Belotelov V (2011). Enhanced magneto-optical effects in magnetoplasmonic crystals. Nat. Nanotechnol..

[2] Lin H, Zhou S, Teng H (2011). Synthesis of Tb_3_Al_5_O_12_ (TAG) transparent ceramics for potential magneto-optical applications. Opt. Mater..

[3] Sun Y (2013). Damping in yttrium iron garnet nanoscale films capped by platinum. Phys. Rev. Lett..

[4] Kajiwara Y (2010). Transmission of electrical signals by spin-wave interconversion in a magnetic insulator. Nature.

[5] Schneider T (2008). Realization of spin-wave logic gates. Appl. Phys. Lett..

[6] Schloemann EF (1988). Circulators for microwave and millimeter-wave integrated circuits. Proc. IEEE.

[7] Rodrigue G (1988). A generation of microwave ferrite devices. Proc. IEEE.

[8] Teurtrie, A. et al. Atmosphere-induced reversible resistivity changes in Ca/Y-doped bismuth iron garnet thin films. *Adv. Funct. Mater.* **29**, 1904958 (2019).

[9] Vasili HB (2017). Direct observation of multivalent states and 4*f*-3*d* charge transfer in Ce-doped yttrium iron garnet thin films. Phys. Rev. B: Condens Matter.

[10] Xu, K. et al. Atomic-scale insights into quantum-order parameters in bismuth-doped iron garnet. *Proc. Natl Acad. Sci. USA* **118**, e2101106118 (2021). 10.1073/pnas.2101106118 PMC8157958 33975955

[11] Qing-Hui Y (2009). Magneto-optical and microwave properties of LuBiIG thin films prepared by liquid phase epitaxy method from lead-free flux. Chin. Phys. Lett..

[12] Zhang D (2019). Effect of lattice mismatch on the laser-induced damage thresholds of (BiTm)_3_(GaFe)_5_O_12_ thin films. Appl Surf. Sci..

[13] Zhang D (2019). Laser-induced damage of garnet films grown by LPE. Opt. Mater..

[14] Hansen P, Witter K (1985). Growth‐induced uniaxial anisotropy of bismuth‐substituted iron‐garnet films. J. Appl Phys..

[15] Rabier J, Veyssière P, Grilhé J (1976). Possibility of stacking faults and dissociation of dislocations in the garnet structure. Phys. Status Solidi (a).

[16] Gutowski MW, Piotrowski K, Gutowska MU, Szewczyk A (2001). Conformal lattice of magnetic bubble domains in garnet film. J. Magn. Magn. Mater..

[17] McKenna KP (2014). Atomic-scale structure and properties of highly stable antiphase boundary defects in Fe_3_O_4_. Nat. Commun..

[18] Chung S-Y, Choi S-Y, Yamamoto T, Ikuhara Y (2008). Atomic-scale visualization of antisite defects in LiFePO_4_. Phys. Rev. Lett..

[19] Wang Z (2018). Designing antiphase boundaries by atomic control of heterointerfaces. Proc. Natl Acad. Sci. USA.

[20] Vronka M, Heczko O, De, Graef M (2019). Influence of antiphase and ferroelastic domain boundaries on ferromagnetic domain wall width in multiferroic Ni-Mn-Ga compound. Appl Phys. Lett..

[21] Singh AV (2017). Bulk single crystal-like structural and magnetic characteristics of epitaxial spinel ferrite thin films with elimination of antiphase boundaries. Adv. Mater..

[22] Muller DA, Nakagawa N, Ohtomo A, Grazul JL, Hwang HY (2004). Atomic-scale imaging of nanoengineered oxygen vacancy profiles in SrTiO_3_. Nature.

[23] Tan H (2011). 2D atomic mapping of oxidation states in transition metal oxides by scanning transmission electron microscopy and electron energy-loss spectroscopy. Phys. Rev. Lett..

[24] Iori F (2019). Bismuth iron garnet: Ab initio study of electronic properties. Phys. Rev. B: Condens Matter.

[25] Pennycook SJ, Jesson DE (1991). High-resolution Z-contrast imaging of crystals. Ultramicroscopy.

[26] Shafieizadeh, Z., Xin, Y., Koohpayeh, S. M., Huang, Q. & Zhou, H. Superdislocations and point defects in pyrochlore Yb_2_Ti_2_O_7_ single crystals and implication on magnetic ground states. *Sci. Rep.* **8**, 17202 (2018). 10.1038/s41598-018-35283-w PMC6249211 30464180

[27] Wei X-K (2014). Ferroelectric translational antiphase boundaries in nonpolar materials. Nat. Commun..

[28] Chlan V (2005). Antisite defects in lutetium and yttrium iron garnets prepared by liquid mix technique. J. Magn. Magn. Mater..

[29] Selim FA, Solodovnikov D, Weber MH, Lynn KG (2007). Identification of defects in Y_3_Al_5_O_12_ crystals by positron annihilation spectroscopy. Appl Phys. Lett..

[30] Przybylińska H (2014). Rare-earth antisites in lutetium aluminum garnets: Influence on lattice parameter and Ce^3+^ multicenter structure. Opt. Mater..

[31] Shannon R (1976). Revised effective ionic radii and systematic study of inter atomic distances in halides and chalcogenides. Acta Crystallogr. Sect. A.

[32] Tian H (2014). Interface-induced modulation of charge and polarization in thin film Fe_3_O_4_. Adv. Mater..

[33] Gloter A (2017). Atomically resolved mapping of EELS fine structures. Mater. Sci. Semicond. Process.

[34] Rojac T (2017). Domain-wall conduction in ferroelectric BiFeO_3_ controlled by accumulation of charged defects. Nat. Mater..

[35] Bencan A (2020). Domain-wall pinning and defect ordering in BiFeO_3_ probed on the atomic and nanoscale. Nat. Commun..

[36] Mishra R (2014). Oxygen-VAcancy-induced Polar Behavior in (LaFeO_3_)_2_/(SrFeO_3_) superlattices. Nano Lett..

[37] Choi S-Y (2015). Assessment of strain-generated oxygen vacancies using SrTiO_3_ bicrystals. Nano Lett..

[38] Shu D-J, Ge S-T, Wang M, Ming N-B (2008). Interplay between external strain and oxygen vacancies on a rutile TiO_2_ (110) surface. Phys. Rev. Lett..

[39] Popova E (2001). Perpendicular magnetic anisotropy in ultrathin yttrium iron garnet films prepared by pulsed laser deposition technique. J. Vac. Sci. Technol. A.

[40] Schaumann G, Völkl J, Alefeld G (1968). Relaxation process due to long-range diffusion of hydrogen and deuterium in niobium. Phys. Rev. Lett..

[41] Li L (2021). Room-temperature oxygen vacancy migration induced reversible phase transformation during the anelastic deformation in CuO. Nat. Commun..

[42] Gorsky W (1935). Theorie der elastischen Nachwirkung in ungeordneten Mischkristallen (elastische Nachwirkung zweiter Art). Phys. Z. Sowjetunion.

[43] Siddons P, Bell NC, Cai Y, Adams CS, Hughes IG (2009). A gigahertz-bandwidth atomic probe based on the slow-light Faraday effect. Nat. Photonics.

[44] Kruk A, Mrózek M (2020). The measurement of Faraday effect of translucent material in the entire visible spectrum. Measurement.

[45] Thersleff T, Rusz J, Hjörvarsson B, Leifer K (2016). Detection of magnetic circular dichroism with subnanometer convergent electron beams. Phys. Rev. B: Condens Matter.

[46] Rose, A. Human Vision. In: Vision: Human and Electronic (ed. Rose, A.) (Springer US, 1973).

[47] Zhang ZH (2009). Evidence of intrinsic ferromagnetism in individual dilute magnetic semiconducting nanostructures. Nat. Nanotechnol..

[48] Schattschneider P (2006). Detection of magnetic circular dichroism using a transmission electron microscope. Nature.

[49] Izumi M, Ogimoto Y, Manako T, Kawasaki M, Tokura Y (2002). Interface effect and its doping dependence in La_1-*x*_Sr_*x*_MnO_3_/SrTiO_3_ superlattices. J. Phys. Soc. Jpn..

[50] Guo EJ (2018). Removal of the magnetic dead layer by geometric design. Adv. Funct. Mater..

[51] Marín L (2015). Observation of the strain induced magnetic phase segregation in manganite thin films. Nano Lett..

[52] Sugiyama J (1997). The effect of oxygen deficiency on the structural phase transition and electronic and magnetic properties of the spinel. J. Phys: Condens Matter.

[53] Dumont Y (2005). Superexchange and iron valence control by off-stoichiometry in yttrium iron garnet thin films grown by pulsed laser deposition. J. Appl Phys..

[54] Anderson PW (1950). Antiferromagnetism. Theory of Superexchange Interaction. Phys. Rev..

[55] Kittel, C. *Introduction to solid state physics*. (John Wiley & Sons, NY, 1976).

[56] Liang X, Xie J, Deng L, Bi L (2015). First principles calculation on the magnetic, optical properties and oxygen vacancy effect of Ce_x_Y_3−x_Fe_5_O_12_. Appl. Phys. Lett..

[57] Song D, Ma L, Zhou S, Zhu J (2015). Oxygen deficiency induced deterioration in microstructure and magnetic properties at Y_3_Fe_5_O_12_/Pt interface. Appl. Phys. Lett..

[58] Gareyeva ZV, Doroshenko RA (2004). Optical absorption of octahedral ions Fe^2+^, Fe^4+^ and photoinduced effect in YIG single crystals. J. Magn. Magn. Mater..

[59] Antonini B (1980). Oxidizing effects of high temperature annealing in reducing atmosphere in Ca-doped YIG films. J. Magn. Magn. Mater..

[60] Tian X, Brennecka GL, Tan X (2020). Structural instability in electrically stressed, oxygen deficient BaTiO_3_ nanocrystals. Adv. Funct. Mater..

[61] Clark GJ, LeGoues FK, Marwick AD, Laibowitz RB, Koch R (1987). Ion beam amorphization of YBa_2_Cu_3_O_x_. Appl. Phys. Lett..

[62] Helseth LE, Kurniawan M, Hansen RW, Johansen TH (2008). Large domain walls near crack lines in ferrimagnetic garnet films. Phys. Rev. B: Condens Matter.

[63] Nistor I, Holthaus C, Mayergoyz ID, Krafft C (2006). Development of liquid phase epitaxy-grown (Bi, Gd, Lu)-substituted thin-film iron garnets. J. Appl. Phys..

[64] Löffler S, Schattschneider P (2010). A software package for the simulation of energy-loss magnetic chiral dichroism. Ultramicroscopy.

[65] Song D, Rusz J, Cai J, Zhu J (2016). Detection of electron magnetic circular dichroism signals under zone axial diffraction geometry. Ultramicroscopy.

[66] Peters JJ (2015). Artefacts in geometric phase analysis of compound materials. Ultramicroscopy.

[67] Kresse G, Furthmüller J (1996). Efficient iterative schemes for ab initio total-energy calculations using a plane-wave basis set. Phys. Rev. B: Condens Matter.

[68] Perdew JP, Burke K, Ernzerhof M (1996). Generalized gradient approximation made simple. Phys. Rev. Lett..

[69] Perdew JP (2008). Restoring the density-gradient expansion for exchange in solids and surfaces. Phys. Rev. Lett..

[70] Blöchl PE (1994). Projector augmented-wave method. Phys. Rev. B: Condens Matter.

