# Supplementary material for: Direct investigation of the atomic structure and decreased magnetism of antiphase boundaries in garnet
Source: Nat Commun. 2022 Jun 9;13:3206. doi: 10.1038/s41467-022-30992-3 (PMC9184601; doi:10.1038/s41467-022-30992-3)
Supplement: Supplementary file 1 — Supplementary Information [file 41467_2022_30992_MOESM1_ESM.pdf]

Supplementary Information for

Direct investigation of the atomic structure and decreased magnetism of antiphase boundaries in garnet

Kun Xu[1,2,3,7], Ting Lin[4,7], Yiheng Rao[5,6], Ziqiang Wang[1], Qinghui Yang[5], Huaiwu Zhang[5], Jing Zhu[1,2,3*]

[1]National Center for Electron Microscopy in Beijing, School of Materials Science and Engineering, Tsinghua University, Beijing 100084, P.R. China

[2]Ji Hua Laboratory

[3]Central Nano & Micro Mechanism, Beijing, Tsinghua University, Beijing 100084, P.R. China

[4]Department of Chemical and Biological Engineering, The Hong Kong University of Science and Technology, Clear Water Bay, Hong Kong SAR, P.R. China

[5]State Key Laboratory of Electronic Thin Films and Integrated Devices, University of Electronic Science and Technology of China, Chengdu 610054, P.R. China

[6]Hubei Yangtze Memory Laboratories, Wuhan 430205, PR China

[7]These authors contributed equally: Kun Xu, Ting Lin.

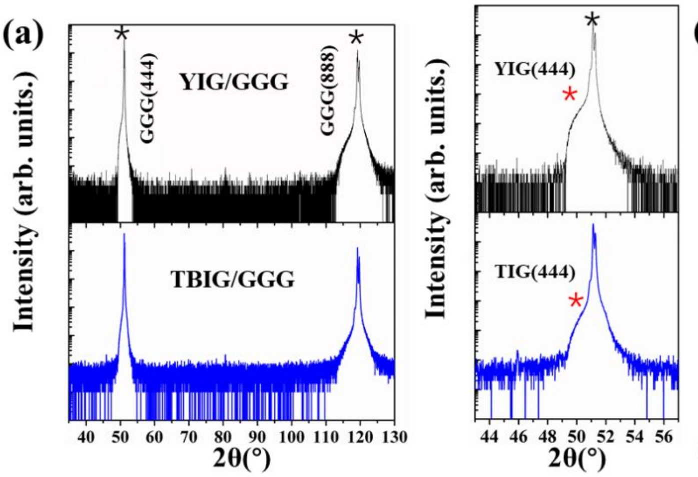

(b)

Element	Atomic Fraction (%)	Atomic Error (%)
O	62.46	5.14
Fe	24.11	3.54
Ga	5.30	0.82
Tm	6.68	1.01
Bi	1.45	0.23

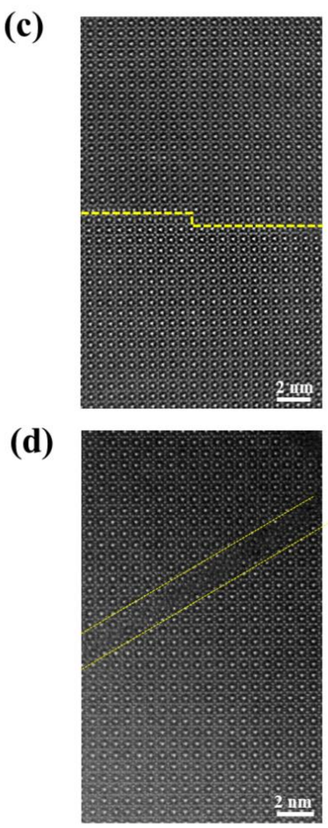

Supplementary Fig. 1. (a) The X-ray diffraction phase analysis of $Y_3Fe_5O_{12}$ (YIG) and TBIG film prepared with growth rate 1.0 μm/min. All show the obvious peaks as (444) and (888), keeping garnet structure as YIG without any extra phases or crystal orientations. (**b**) Quantitative contents of different element in TBIG films were measured by EDS. (**c**), (**d**) STEM-HAADF images with lower magnification in APB-I and APB-II. it shows that these APBs have sharp interface between adjacent domains.

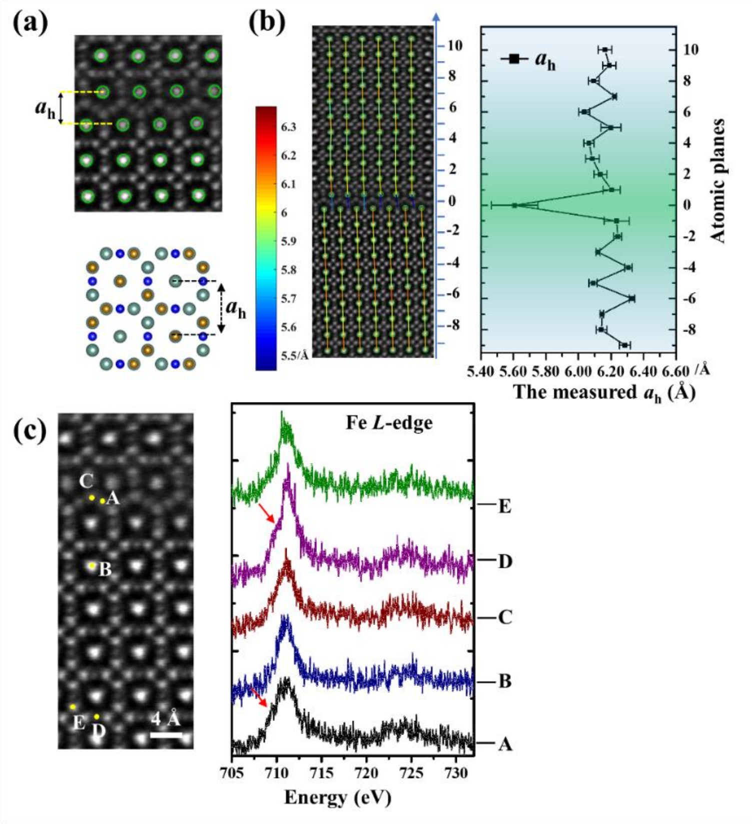

Supplementary Fig. 2. The measured half lattice constants and site-specific EELS across the interface of APB-I. (**a**) STEM-HAADF image of APB-I, where central brightest atom columns are indexed by green circles. And defined half lattice constants along [100] direction are indicated by arrows, as illustrated in atomic model. two-dimensional Gaussian fitting method is applied to determine the positions of atomic columns indexed by green circles. (**b**) The map of a_h constant is shown in the left side. The measured lattice constants are plotted across the interface of APB-I in the right side. The error bar is defined by the standard deviation of measured spacing a_h. It reveals that a_h lattice constant in the interface of APB-I decreases obviously, when compared to that away from interface. (**c**) Fe L_3 edges in position A and D show clear shoulder-like feature marked by red arrows, which corresponding to the octahedral coordination.

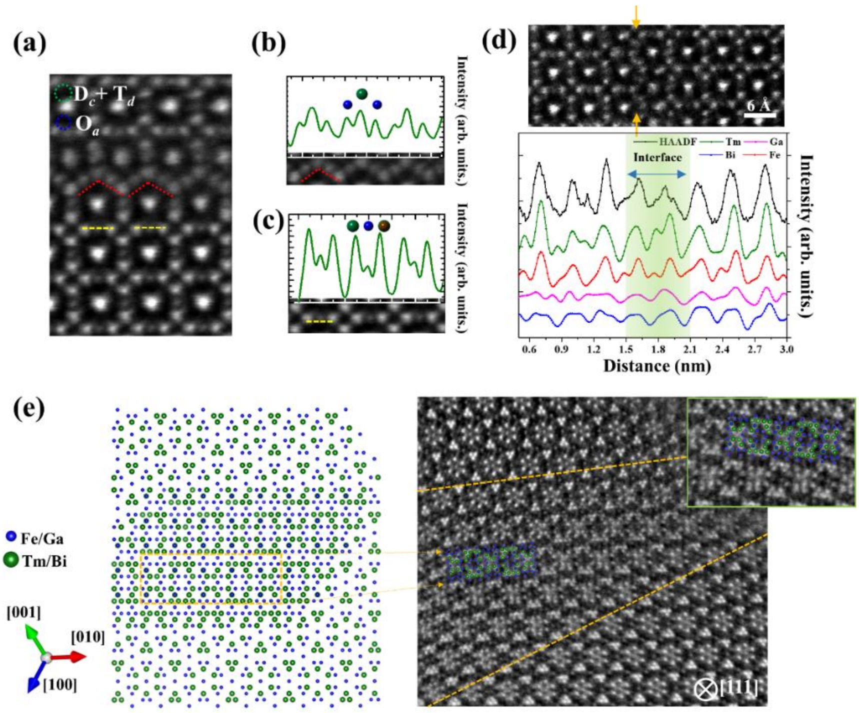

Supplementary Fig. 3. Atomic column intensity, EDS intensity profile across the interface of APB-I and atomic structure along [111] zone axis . (**a**) STEM-HAADF images of interfacial regions, where red lines identify the zigzag structure and yellow lines identify the normal structure. (**b**), (**c**) the intensity profile of atomic columns extracted from the region in (**a**) marked by red lines and yellow lines across the interface. It can be found the intensities of different atoms in zigzag structure is opposite to that in normal structure. (**d**) The EDS intensity profile across the interface. It indicates that different elements are distributed randomly and uniformly across the interface. (**e**) Atomic structure of APB-I along [111] zone axis. Experimental HAADF image across the APB-I along [111] zone axis is presented in the left side, where two adjacent regions close to APB-I are separated by orange lines. Magnified HAADF image from APB-I is consistent with interfacial structure model marked by orange rectangle in the right side. By employing the proposed atom model, the projected atomic structure along [111] zone axis is shown in the left side, accordingly. The experimental atomic image is well consistent with projected atomic model.

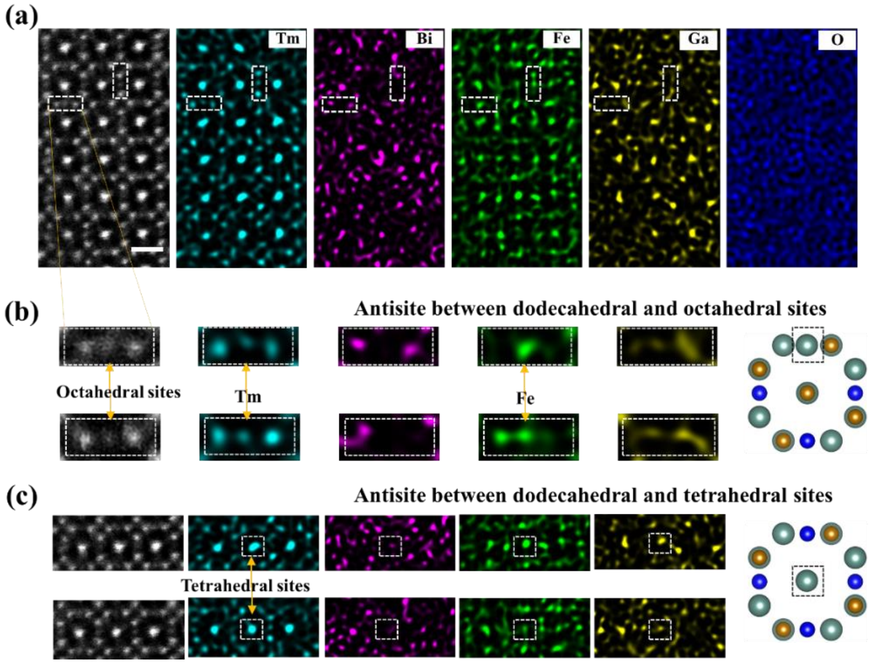

Supplementary Fig. 4. The EDS maps of different elements in the region close to APB-I. (**a**) Atomic scale EDS maps of Tm, Bi, Fe, Ga, O, respectively. Different colors represent different elements. The intensity of color represents the concentration of elements. (**b**) The enlarged EDS maps extracted from the regions highlighted by white rectangles in (**a**). The octahedral sites in the maps are marked by orange arrows, and obvious signals of Tm are observed in the octahedral sites. The observation of site occupation indicates that Tm elements could partially occupy in octahedral sites due to this antisite behavior. **(c)** The tetrahedral sites in the maps are marked by orange arrows. Although tetrahedral sites and dodecahedral sites are overlapped along [001] zone axis. It can be found that the positions indicated by yellow arrows in the lower panel show decreased signals of Fe and Ga elements, compared with positions in the upper panel. This indicates that Tm elements could also occupy in tetrahedral sites due to this antisite behavior.

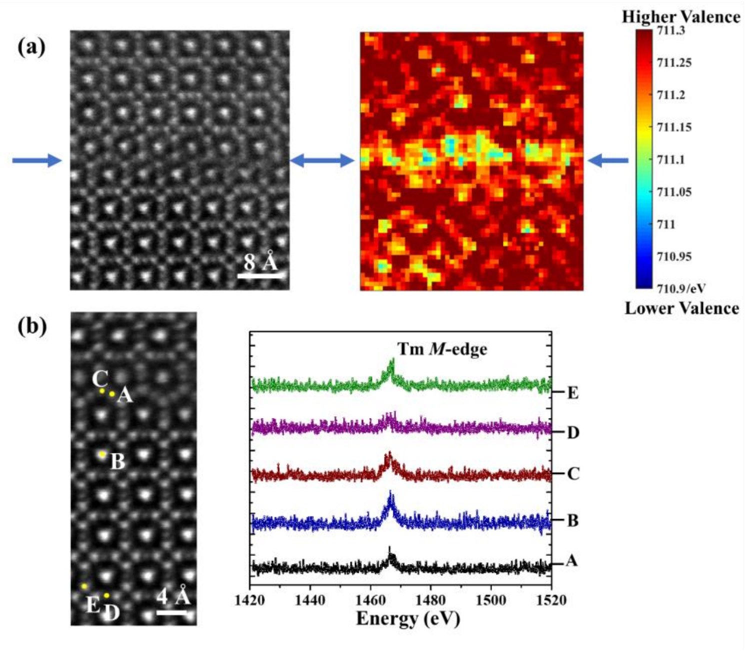

Supplementary Fig. 5. EELS analysis of the interface and region close to APB-I at atomic scale. (**a**) The positions of Fe L_3 edge are extracted from EELS across the APB-I at atomic scale. (**b**) Electron energy loss spectra (EELS) at atomic scale are acquired across the interfacial region. On the right side, EELS of Tm M_5 edge in different sites labeled by point A, B, C, D and E are extracted as plots in series.

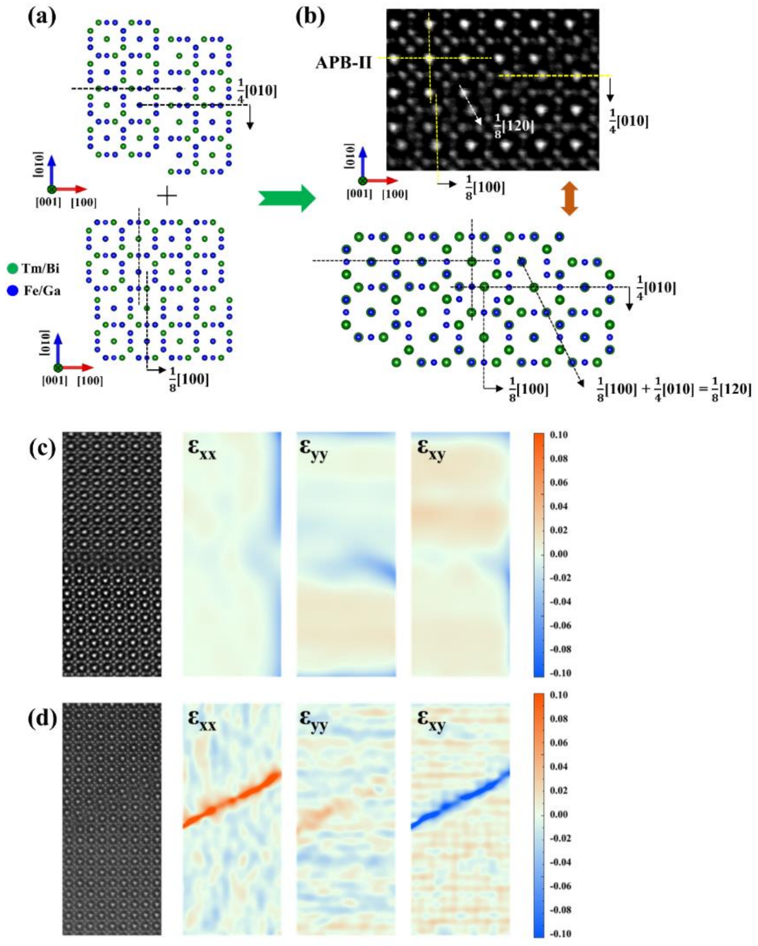

Supplementary Fig. 6. (**a**) Two adjacent domains shift against each other along horizontal and vertical directions respectively, by 1/4[010] and 1/8[100] unit cell. (**b**) When these two shifting occurs together, it can lead to the additive shifting, equal to 1/8[120], arising from horizontal shifting by 1/8[100] and vertical shifting by 1/4[010]. (**c**) The strain maps of APB-I by geometric phase analysis. (**d**) The strain maps of APB-II. To compare these strain maps of two APBs, we can find that the strain along APB-II is larger than that of APB-I.

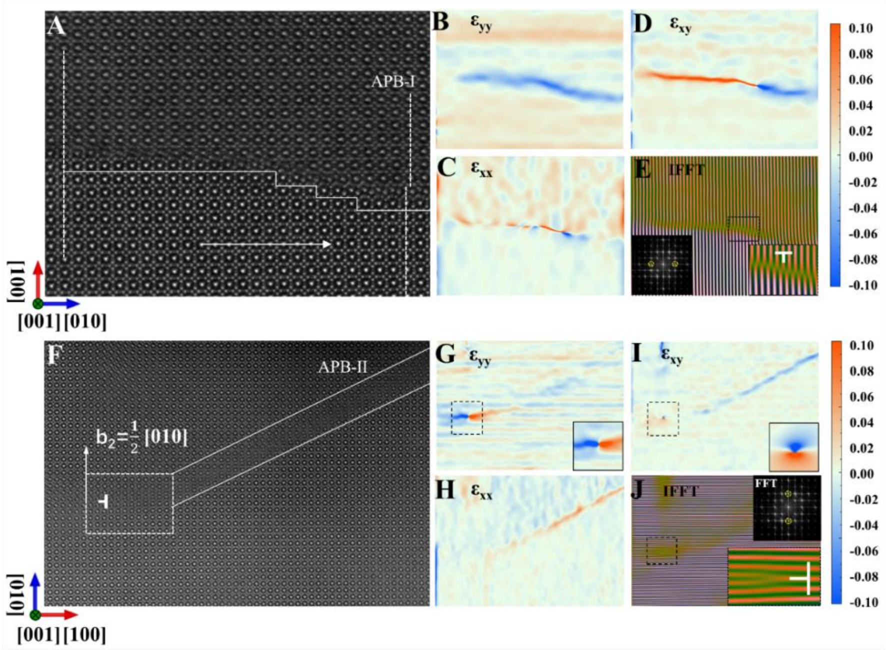

Supplementary Fig. 7. **The defective region related to the formation of APBs.** (**A**), (**F**) STEM-HAADF images of region in low magnification, including defective area close to the APBs, white lines in (A) mark the interface as steps along the direction indexed by arrows. (**B**), (**C**), (**D**) and (**G**), (**H**), (**I**) GPA component ε_{yy}, ε_{xx} and ε_{xy} corresponding to the defective region of (A) and (F). (**E**) The reconstructed image of the (400) plane marked by yellow circle in inset after an Inverse Fast Fourier Transformation (IFFT). The inset in enlarge view indicates the planar misfits along the steps. (**J**) The reconstructed image of the (040) plane marked by yellow circle in inset after an Inverse Fast Fourier Transformation (IFFT). The inset in enlarged view indicates the edge dislocation with Burgers vector $\boldsymbol{b_2}$ = 1/2[010].

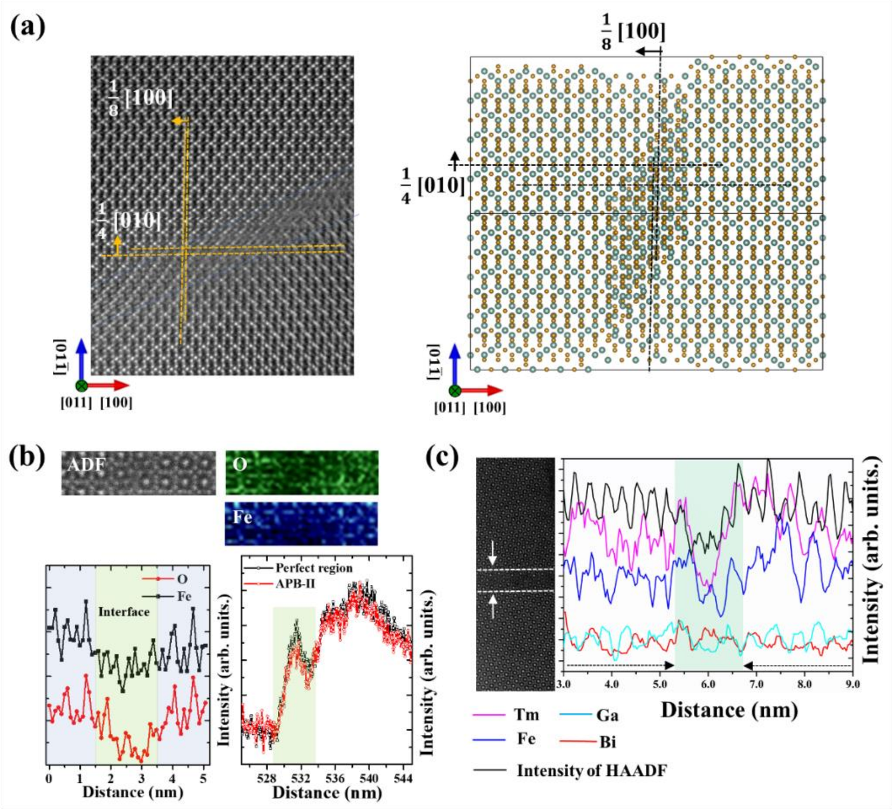

Supplementary Fig. 8. Atomic structure and composition analysis of APB-II. (**a**) STEM-HAADF images of APB-II along [011] zone axis, it shows clear antiphase boundaries along vertical and horizontal directions. The shifting along two directions is consistent with the result along [001] direction. (**b**) EELS map of Fe and O elements, and signals of Fe and O extracted from EELS map are plotted as the function of distance across the APB-II. Different colors represent different elements. The intensity of color represents the concentration of elements. O-*K* edge spectra in different region are compared to show different pre-peak intensity, indicating the existence of oxygen vacancies in APB-II. (**c**) The profiles of EDS intensity of different compositional elements are plotted across the interface.

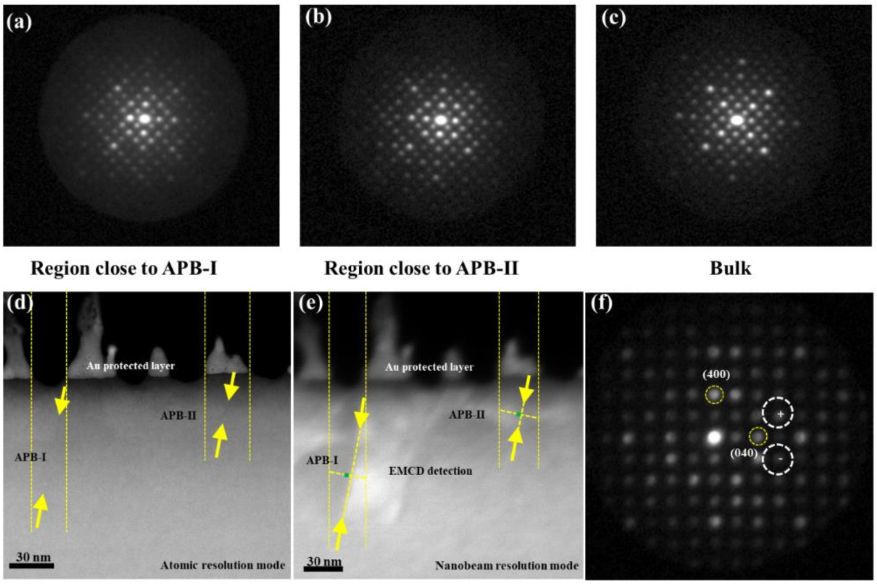

Supplementary Fig. 9. Locating the positions of two types Antiphase boundaries (APBs) in both atomic resolution and nanobeam scanning transmission electron microscopy (STEM) mode precisely. (**a**), (**b**), (**c**) STEM-nanobeam diffraction corresponding to the different regions. (**d**) STEM-HAADF image at atomic resolution mode. (**e**) STEM-HAADF image at nanobeam resolution mode. Yellow lines are used to locate the positions of APBs precisely in both imaging mode. Green dots are used to mark the positions where EMCD detections are conducted. (**f**) Electron diffraction under nanobeam mode. Circles in maps represents positions of EELS collection apertures in diffraction plane. By moving the collection apertures in these conjugated positions, we can acquire positive and negative EEL spectra.

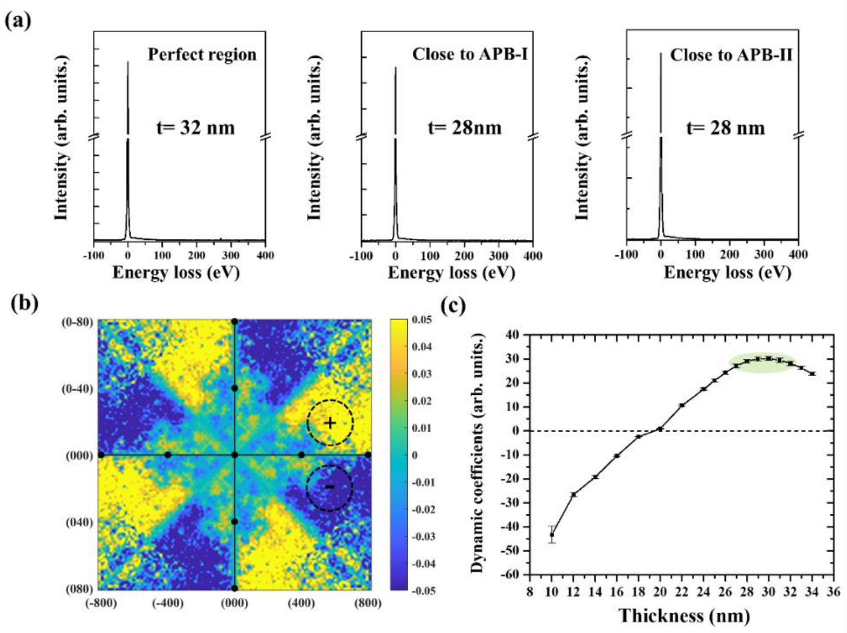

Supplementary Fig. 10. EMCD dynamic coefficient distribution under [001] zone axis. (**a**) Thickness of regions where EMCD signals are collected, are measured by low loss EELS. (**b**) Distribution of EMCD dynamic coefficients with thickness of 28 nm under [001] zone axis. Circles in maps represents positions of collection apertures in diffraction plane. The color represent the EMCD dynamic coefficients with arbitrary units. By moving the collection apertures in these conjugated positions, we can acquire positive and negative spectra. (**c**) Relative dynamic coefficients of EMCD are plotted as function of thickness at 300 kV for collection positions as described in (b).

Supplementary Note 1. Signal to noise ratio (SNR) analysis:

we can calculate the signal to noise ratio (SNR) of the EMCD signal independently on the Fe L_3 and L_2 edge. To quantitatively determine the confidence level of electron magnetic circular dichroism (EMCD) spectrum, this can be realized by fitting a combination of two Gaussian peaks to the EMCD signals,[53] which can be expressed by using the following equation:

$$f(E) = A_3 e^{-(\frac{E-\mu_3}{\sigma_3})^2} + A_2 e^{-(\frac{E-\mu_2}{\sigma_2})^2} \quad \text{Supplementary Eq. (1)}$$

Here, E represents the independent variable of electron energy loss (eV), μ the central position of both $L_{2,3}$ peaks. Subscripts 2 and 3 refer to the Fe L_2 and L_3 edges, respectively. From the EELS spectra of Fe $L_{2,3}$, we can find that μ_2 and μ_3 are constrained to lie between 720-725 eV for L_2 edge and 706-715 eV for L_3. σ is related to the width of peaks in both edges. And due to the opposite intensity of two peaks for EMCD spectra, A_3 is constrained to take on the opposite sign from A_2. The signal is defined as the value of parameters A for both peaks individually. An estimate of the noise (root mean value) is obtained by computing the standard deviation of the fit residuals in the range 660–760 eV. SNR is then defined as the ratio between the signal and this noise estimation. The total number of primary electrons contributing to the EMCD signal for both L_3 and L_2 was calculated by integrating the individual fitting curves. The confidence level[54] could be estimated based on the probability distribution of a noisy quantity about its mean value, which can be expressed by equation (2) (probability distribution of a noisy quantity can be seen as shown in Figure 1-1):

$$p = (\frac{1}{2\pi})^{1/2} e^{(-\frac{k^2}{2})} \quad \text{Supplementary Eq. (2)}$$

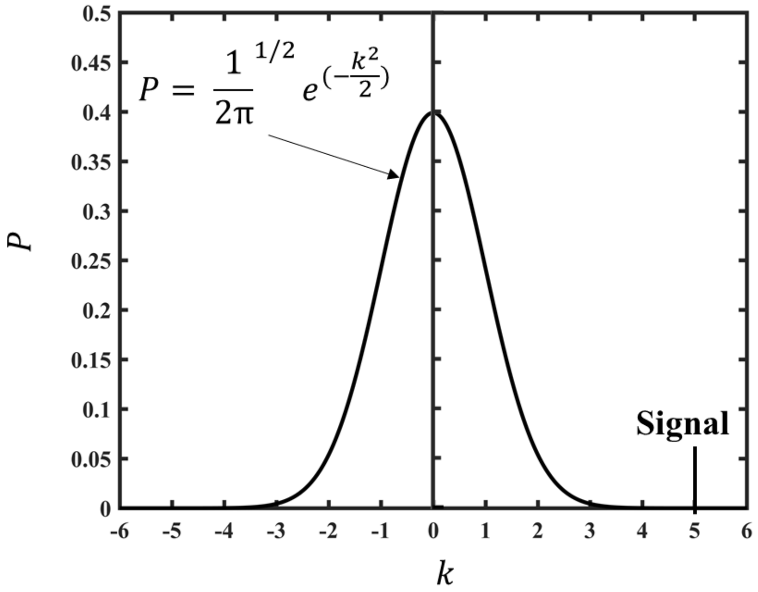

Supplementary Note Figure 1-1. Probability distribution of a noisy quantity about its mean value.

Here k refers to signal to noise ratio (SNR). p refers to probability of a noisy quantity about its mean value. 1- p represents the confidence level of signal. When SNR exceed 5 by applying the Rose criterion, it means that a confidence level will be almost 99.9999%, indicating the signal is quite

credible.[54]

(1) For Fe EMCD spectra recorded from the perfect region (Figure 4a in main text of manuscript), the parameters are fitted as follows:

Coefficients (with 95% confidence bounds for Gaussian fitting):
A_3 = 1160.1 (dimensionless quantity); μ_3 = 709.8 eV;
A_2 = -399.1 (dimensionless quantity); μ_2 = 721.9 eV

Noise estimation : 137.2 (dimensionless quantity)
SNR on the L_3 edge : 1160.1/137.2 = 8.46 (confidence level > 99.9999%);
SNR on the L_2 edge : 399.1/137.2 =2.91 (confidence level = 99.42%);

(2) For Fe EMCD spectra recorded from the region close to the APB-I (Figure 4b in main text of manuscript), the parameters are fitted as follows:
A_3 = 167.3 (dimensionless quantity); μ_3 = 709.2 eV
A_2 = -108.9 (dimensionless quantity); μ_2 = 723.1 eV

Noise estimation : 122.3 (dimensionless quantity)
SNR on the L_3 edge : 167.3/122.3 = 1.37 (confidence level =84.39%);
SNR on the L_2 edge : 108.9/122.3 =0.890 (confidence level =73.16%);

(3) For Fe EMCD spectra recorded from the region close to the APB-II (Figure 4c in main text of manuscript), the parameters are fitted as follows:
A_3 = 53.8 (dimensionless quantity); μ_3 = 709.5 eV
A_2 = -71.8 (dimensionless quantity); μ_2 = 723.9 eV

Noise estimation : 79.44 (dimensionless quantity)
SNR on the L_3 edge : 53.8/79.44 = 0.677 (confidence level =68.28%);
SNR on the L_2 edge : 71.8/79.44 =0.904 (confidence level =73.48%);

Supplementary Note Table 1. Signal to noise ratio analysis for Figure 4 in main text of manuscript.

Investigated regions	SNR(L_3)	Confidence level (L_3)	SNR(L_2)	Confidence level (L_2)
Internal region	8.46	> 99.9999%	2.91	99.42%
Region close to APB-I	1.37	84.39%	0.890	73.16%
Region close to APB-II	0.677	68.28%	0.904	73.48%

After reviewing these confidence level analyses of EMCD signal, we can find that confidence level of EMCD signal in perfect regions is quite credible, while EMCD signals in regions close to the APBs have almost the same order of magnitude when compared with noise fluctuation. Therefore, it is evident that EMCD signals of regions close to the APBs decreased when compared to the EMCD signal of regions close to the APBs. These further analyses can strongly support our discussion.

Besides, we perform the repeatable EMCD experiments under the same condition. The details can be seen below.

Repeatable EMCD experimental results:

(1) For Fe EMCD spectra recorded from the perfect region, the parameters are fitted as follows:

Coefficients (with 95% confidence bounds for Gaussian fitting):
A_3 = 872.1 (dimensionless quantity); μ_3 = 708.5 eV
A_2 = -235.5 (dimensionless quantity); μ_2 = 723.1 eV

Noise estimation : 134.4 (dimensionless quantity)
SNR on the L_3 edge : 872.1/134.4 = 6.49 (confidence level > 99.9999%);
SNR on the L_2 edge : 235.5/134.4 =1.75 (confidence level = 91.41%);

(2) For Fe EMCD spectra recorded from the region close to the APB-I, the parameters are fitted as follows:
A_3 = -39.5 (dimensionless quantity); μ_3 = 708.2 eV
A_2 = -106.2 (dimensionless quantity); μ_2 = 721.0 eV

Noise estimation : 230.1 (dimensionless quantity)
SNR on the L_3 edge : 39.5/230.1 = 0.172 (confidence level =60.68%);
SNR on the L_2 edge : 106.2/230.1 =0.462 (confidence level =64.11%);

(3) For Fe EMCD spectra recorded from the region close to the APB-II, the parameters are fitted as follows:
A_3 = 16.8 (dimensionless quantity); μ_3 = 708.8 eV;
A_2 = 25.9 (dimensionless quantity); μ_2 = 722.2 eV

Noise estimation : 136.3 (dimensionless quantity)
SNR on the L_3 edge : 16.8/136.3 = 0.123 (confidence level =60.41%);
SNR on the L_2 edge : 25.9/136.3 =0.190 (confidence level =60.82%);
The Gaussian fitting curve for the Fe EMCD spectra is shown in Figure 1-2.

Supplementary Note Table 2. Signal to noise ratio analysis for repeated EMCD experiment

Investigated regions	SNR(L_3)	Confidence level (L_3)	SNR(L_2)	Confidence level (L_2)
Internal region	6.49	> 99.9999%	1.75	91.41%
Region close to APB-I	0.172	60.68%	0.462	64.11%
Region close to APB-II	0.123	60.41%	0.190	60.82%

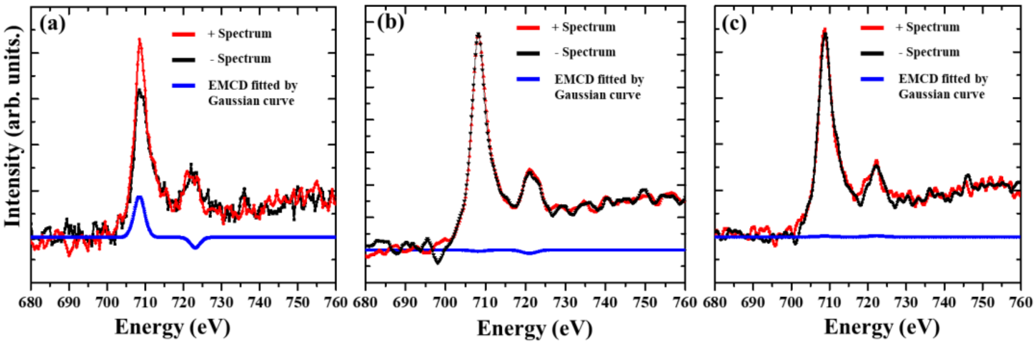

Supplementary Note Figure 1-2. Repeated EMCD experiment under the same experiment condition. Fe EMCD spectra recorded from the perfect region (a), close to the APB-I (b), close to the APB-II (c) . The Gaussian fitting curve for the Fe EMCD spectra is plotted by blue line.

In our repeated experiment, the resulting SNR and confidence levels for Fe EMCD by using Gaussian fitting did not change significantly. So, the results can be repeated in EMCD signal of different regions.

Supplementary Table 1. The calculated magnetic moments, bond lengths and bond angles between Fe octahedral and tetrahedral sites, by using density function theory (DFT) calculation. The perfect thulium iron garnet structure (a) and thulium iron garnet structure with 6.25% oxygen vacancies (b) are compared respectively. The calculated magnetic moments, bond lengths and angles in these Fe sites with specific oxygen vacancies are calculated in table, showing larger bonding lengths and decreased angles than that of perfect structure without oxygen vacancies. Besides, the magnetic moments reduce obviously when oxygen vacancies are introduced. The averaged magnetic moment in perfect structure is measured to be 0.790 μ_B/Fe, but can reduce to 0.698 μ_B/Fe when only 6.25% oxygen vacancies is included. In our cases, the oxygen vacancies lead to reduced Fe^{2+} in these regions close to APBs. So, it can be estimated to be less than 0.698 μ_B/Fe, although more oxygen vacancies cannot be included in iron garnet structure, due to the limited capacity for larger supercell with enormous atoms. Besides, the elongated Fe_{oct}-O-Fe_{tet} Bond lengths and decreased Fe_{oct}-O-Fe_{tet} angles will further reduced super-exchange interaction in two different magnetic sublattice when more oxygen vacancies are included.

(a)

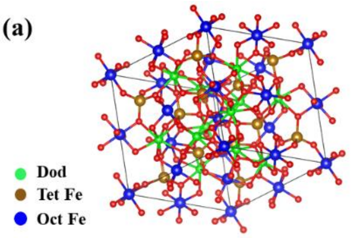

Fe sites	Magnetic moments (μ_B)	Fe_{oct}-O-Fe_{tet} Bond lengths (Å)	Fe_{oct}-O-Fe_{tet} Angle (°)
$Fe_{octahedron}$	-4.107	3.4570	124.61
$Fe_{tetrahedron}$	4.188	3.4570	124.61

(b)

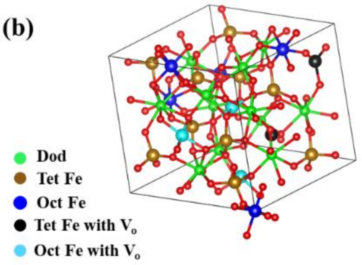

Fe sites	Magnetic moments (μ_B)	Fe_{oct}-O-Fe_{tet} Bond lengths (Å)	Fe_{oct}-O-Fe_{tet} Angle (°)
$Fe_{octahedron}$ with one V_o	-3.628<br>-3.629	3.4728	120.9728<br>120.8460<br>117.7300
$Fe_{tetrahedron}$ with one V_o	3.623	3.6282	139.2896
$Fe_{tetrahedron}$ with two V_o	1.021	3.6806<br>3.9900	119.4006<br>118.3800<br>116.1200

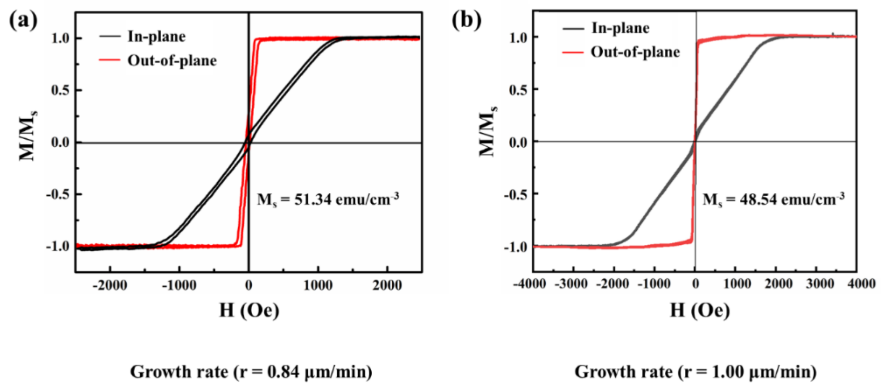

Supplementary Fig. 11. Macroscopic magnetization analysis at room temperature ~ 300 K. **M**(**H**) hysteresis loops of films prepared with a growth rate of 0.84 μm/min (**a**) and 1.00 μm/min (**b**). The saturation magnetization of films prepared with a growth rate of 0.84 μm/min and 1.00 μm/min are 51.34 emu/cm^3 and 48.54 emu/cm^3 respectively.

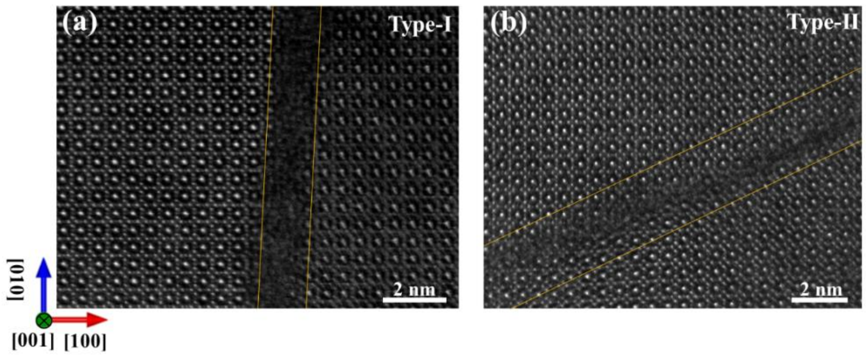

Supplementary Fig. 12. STEM-HAADF images of region with APB-I (**a**) and APB-II (**b**) after being irradiated by ion beam (2kV, 150 pA, Ar^+).
